# *De novo* protein conformational sampling using a probabilistic graphical model

**DOI:** 10.1038/srep16332

**Published:** 2015-11-06

**Authors:** Debswapna Bhattacharya, Jianlin Cheng

**Affiliations:** 1 Department of Computer Science, University of Missouri, Columbia, MO 65211, USA; 2 Informatics Institute, University of Missouri, Columbia, MO 65211, USA; 3 Bond Life Science Center, University of Missouri, Columbia, MO 65211, USA

## Abstract

Efficient exploration of protein conformational space remains challenging especially for large proteins when assembling discretized structural fragments extracted from a protein structure data database. We propose a fragment-free probabilistic graphical model, FUSION, for conformational sampling in continuous space and assess its accuracy using ‘blind’ protein targets with a length up to 250 residues from the CASP11 structure prediction exercise. The method reduces sampling bottlenecks, exhibits strong convergence, and demonstrates better performance than the popular fragment assembly method, ROSETTA, on relatively larger proteins with a length of more than 150 residues in our benchmark set. FUSION is freely available through a web server at http://protein.rnet.missouri.edu/FUSION/.

Successfully predicting protein three-dimensional structures of near-experimental accuracy from their amino acid sequence requires efficient navigation of astronomically large conformational space^1^ accessible to proteins. Fragment assembly approaches^2^,^3^,^4^ include rapid exploration of conformational space by restricting local conformations (i.e., fragments) to those observed in experimentally-solved structures extracted from the Protein Data Bank (PDB), and assembling the fragments to form complete structures. Such a locally restrained search strategy has proven to be an extremely powerful method to fold small proteins (<100 residues) with reasonable accuracy^5^. For larger proteins, however, the inherent rigidity and incomplete coverage of these discrete fragments often impose kinetic limitations in sampling^6^,^7^, hindering the possibility of accurate *de novo* protein structure prediction.

Significant progress has been made recently to overcome the limitations of fragment-based methods by performing probabilistic sampling guided by local structural preferences^8^,^9^,^10^. Although promising and mathematically attractive, these approaches are either based on coarse-grained (i.e., C_α_) representations of protein structure^8^,^9^, or they assume ideality in backbone planarity^10^ (i.e., ω-angles). A coarse-grained model is of limited use in high-resolution protein structure prediction because of the one-to-many correspondence between C_α_ traces and full atomic detail of a protein’s backbone. Also, small, but cumulative, and often systematic, deviations from ideality in backbone planarity exists^11^, which, if ignored, might also lead to possible minor distortions in the structure. Assuming ideal bond lengths and bond angles, the minimum angular degrees of freedom needed are three dihedral angles (ϕ, ψ, ω) to accurately place backbone Cartesian coordinates (x, y, z of three atoms - N, C_α_, and C) of a residue. This is the granularity used by typical fragment assembly methods, such as ROSETTA^2^; however, fragment-free *de novo* sampling at this grain has not yet been demonstrated, to our knowledge.

Here, we propose an Input-Output Hidden Markov Model^12^ (IOHMM) to capture the preferences of the dihedral angles associated with protein backbone (ϕ, ψ, ω) given its sequence as shown in Fig. 1a. An IOHMM is based on a non-homogeneous Markov chain, where emission and transition probabilities depend on the input. IOHMM is, therefore, an appropriate choice for protein structure prediction, where the goal is to sample protein conformation given its sequence (i.e., input). The proposed model, FUSION, captures local relationships between protein sequence and structural features and allows for probabilistic sampling of conformational space of the protein backbone in full-atomic detail (i.e., at the same granularity as fragment assembly) from a continuous space different from the discrete space of fragment assembly.

## Results

In this section, we first briefly describe the architecture of FUSION, then describe sampling strategies, and finally present an evaluation of its performance from various perspectives.

### Architecture of FUSION

**Figure 1:**
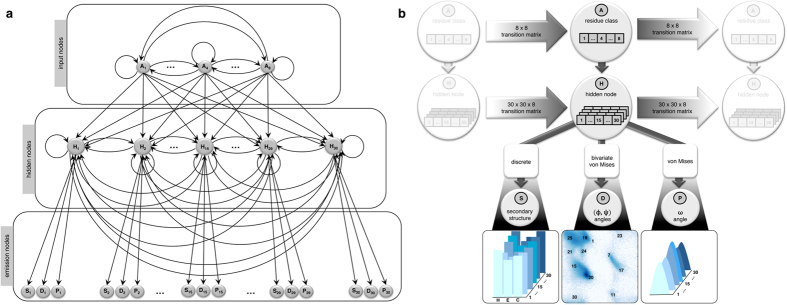
Architecture of FUSION IOHMM. **(a)** Circular nodes represent stochastic variables and arrows in the graph specify the conditional independent relationships among variables. Input nodes capture protein’s sequence space while output (i.e., emission) nodes correspond to structural space. Hidden nodes specify dependencies between sequence and structural space along the sequence (i.e., not only between consecutive nodes). **(b)** In each slice, an input node controls transition of the residue classes in the amino acid sequence (**A**) and a Markov chain of hidden nodes (**H**) captures the sequential dependencies along the peptide chain where each hidden node corresponds to three kinds of emission distributions: (1) three-state secondary structure labels (**S**): helix (H), strand (E), and coil (C), (2) backbone (ϕ, ψ) dihedral angle pairs (**D**), and (3) ω angles associated with peptide bonds (**P**). The type of emission distribution is specified in square boxes.

FUSION ensures sequential dependencies between protein sequence (input) and structural space (output) through a Markov chain of hidden states. In each slice, as presented in Fig. 1b, an input node (**A**) captures the protein’s sequence space. Connections between the input nodes represent the transition probabilities between residues along the protein chain. Output (i.e., emission) nodes correspond to structural space, modeled using secondary structure (**S**), dihedral angle pair (**D**: ϕ, ψ), and peptide bond conformation (**P**: ω). The hidden node (**H)** is a discrete node that can adopt 30 states (which is the optimal number of states) where, each of these states specifies which mixture component is chosen among the possible emission distributions. The optimal number of hidden states and all other associated parameters were determined by training the model using a maximum likelihood method on a large set of representative experimentally-solved protein structures.

### Generating protein conformation

In the trained model, each hidden node value is associated with preferences of secondary structure types, and backbone geometry, conditioned on sequence. This provides a convenient way of generating protein structural features compatible with its sequence. For a given protein sequence, a corresponding hidden node sequence can be sampled from one end to the other through plausible paths in the transition matrices of input and hidden nodes. After obtaining a particular sequence of hidden node values, emission values for the output nodes are drawn from the corresponding conditional probability distributions. It is also possible to seamlessly resample random-length segments of the protein using the forward-backtrack algorithm^13^. Furthermore, the inclusion of secondary structure information into the model allows for sampling the conformational space associated with both amino acid, and, optionally, secondary structure, when the latter becomes available (e.g., predicted from amino acid sequence). The sampled dihedral angles (ϕ, ψ, ω), conditioned on sequence-based observations can then be readily converted into Cartesian coordinates, giving rise to a protein backbone in full-atomic detail. Repeated resampling of random stretches of dihedral angles in FUSION mimic fragment replacement in fragment assembly methods, but in a probabilistic way, which reduces intrinsic sampling bottlenecks imposed by a discretized fragment library (e.g., boundary effects^2^).

The probabilistic nature of FUSION facilitates its effective integration as a proposed distribution in Markov Chain Monte Carlo (MCMC) simulations, under the control of an empirical force field. We used the classic Metropolis-Hastings MCMC approach^14^, by resampling random stretches (3 to 15 residue segment) of the current candidate structure, **x**, having a dihedral angle sequence **d**, to propose a new sequence of dihedral angles **d**′, resulting in the next candidate structure, **x**′, and accepting or rejecting the move using standard Metropolis-Hastings acceptance criterion. Simulations were carried out using the low-resolution scoring function of ROSETTA^15^, together with ambiguous sequence-derived predicted information. FUSION’s model-based conditional sampling approach removes a major bottleneck of using fragment assembly as a proposal distribution that, by contrast, implicitly introduces a system-specific bias into the force field, which is difficult to quantify^16^. Thus, it is generally impossible to satisfy the condition of detailed balance^14^, which is a fundamental prerequisite to ensure that simulations sample the Boltzmann distribution of the applied force filed.

### Blind assessment of FUSION

We blindly tested the generality and accuracy of FUSION using 42 protein targets with a sequence length less than 250 residues that were simultaneously under investigation in X-ray crystallography or NMR spectroscopy laboratories during the 11th community-wide experiment on the Critical Assessment of Techniques for Protein Structure Prediction (CASP11). A reduced representation of protein structure was adopted that used the backbone atoms and a side-chain centroid to generate up to 10,000 low-resolution models for each protein sequence within a limited prediction window of three days. The reduced models were then expanded by adding side chains using the smoothed backbone-dependent rotamer library^17^,^18^ to produce all-atom decoys.

### Angular preferences

**Figure 2:**
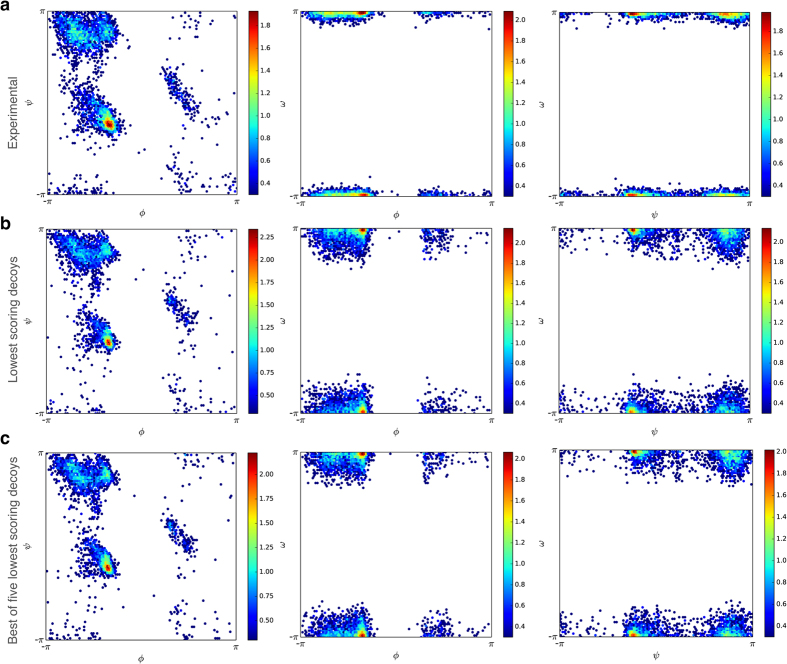
Distributions of dihedral angles ϕ, ψ, and ω. The joint histograms of (ϕ, ψ), (ϕ, ω), and (ψ, ω) are shown for experimental structures (**a**), FUSION-generated decoys with lowest DFIRE score (**b**) and decoys closest to their corresponding experimental structures among the five lowest scoring decoys (**c**), with color code ramping from blue to red for low to high density.

To investigate whether FUSION captures the angular preference of dihedral angles (ϕ, ψ, ω) observed in proteins, we ranked the all-atom decoy population for each target using DFIRE^19^ statistical potential and compared the joint histograms of (ϕ, ψ), (ϕ, ω), (ψ, ω) angles from the lowest scoring decoy as well as the lowest C_α_-rmsd (root mean square deviation of alpha-carbon coordinates after optimal structural superposition) decoy in the set of the top five low-scoring decoys (i.e., best of five) with that of their experimental structures. As shown in Fig. 2, the distribution of (ϕ, ψ) angles was in close agreement with the observations in both cases and covered the entire allowed space of the Ramachandran plot^20^. The distributions of (ϕ, ω) and (ψ, ω), despite correctly capturing the major peaks, revealed noticeable deviations in ω angles compared to their experimental counterparts. However, these apparent outliers might be due to the restraints imposed by the use of ideal bond lengths and angles during the simulations, to some degree.

### Secondary structure propensity

In addition to capturing the dihedral angle distribution, FUSION decoys revealed excellent similarity in overall secondary structure content compared to the experimental structures. In Table 1, we present the secondary structure content of the experimental structures and FUSION decoys. Over the entire benchmark set, having ~34% helix (α-helix, 3_10_-helix, and π-helix) and ~30% β-strand (extended strand, and isolated β-bridge), both lowest scoring and the best of five decoys contained ~32% helix and ~23% β-strand. It should be noted, however, that formation of β-strand residues requires specific nonlocal interaction (i.e., hydrogen bonding), which is beyond the scope of a Markovian model like FUSION, and was primarily achieved by the scoring function.

### Nature of sampled energy landscape

**Figure 3:**
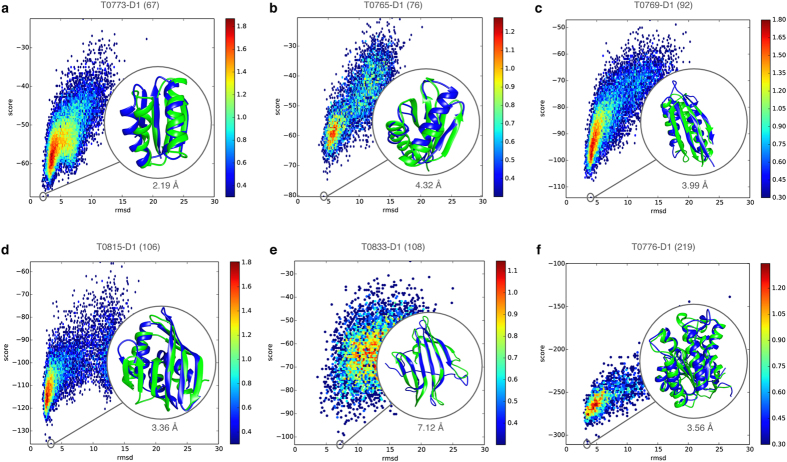
Energy landscapes of FUSION simulations for diverse protein targets. (**a**–**f**) Energy (DFIRE score) verses accuracy (C_α_-rmsd) for decoys produced by FUSION for protein targets T0773-D1 (**a**), T0765-D1 (**b**), T0769-D1 (**c**), T0815-D1 (**d**), T0833-D1 (**e**) and T0776-D1 (**f**), with color coding from blue to red for low to high density and a measure for the underlying energy scale. The lowest scoring decoys (in blue) overlaid on the experimental structures (in green) are highlighted in the insets, with their accuracies (C_α_-rmsd) quantified below. In each case, protein length is indicated within parentheses next to the target name.

To study the energy landscape encountered during FUSION simulations, we examined the relationship between DFIRE energy score and C_α_-rmsd of decoy populations. In Fig. 3, we show 2-dimensional distribution of conformations as a function of the DFIRE energy score, on the *y* axis and the C_α_-rmsd to the native state on the x axis for a diverse set of targets with different topologies and sequence lengths. Strong convergence was observed in several cases as defined by a distinct funnel-shaped energy landscape. FUSION produced convergent sampling across a broad spectrum of target lengths ranging from small targets with a relatively simple fold (like T0773-D1; Fig. 3a) to larger protein having complex topologies (like T0776-D1; Fig. 3f). The energy landscape encountered by FUSION over the entire benchmark set is presented in Supplementary Fig. 1.

### Extent and distribution of conformational sampling

To further examine the degree of conformational sampling done by FUSION, we investigated the proportion of good decoys (having a C_α_-rmsd below 6 Å with the native structure), the accuracies of the decoys having the lowest DFIRE scores, and the best decoys out of the five lowest DFIRE scores. Table 2 reports each of these measures for all the targets in the benchmark set. For 24 out of 42 targets, FUSION generated some good decoys with C_α_-rmsd less than or equal to 6 Å. For 15 targets, the best of the top five lowest scoring decoys selected by DFIRE from all the decoys generated by FUSION had an accuracy better than 6 Å, even though the percentage of good decoys is not always high. As expected, smaller size targets tend to have a much higher proportion of good decoys as well as a higher accuracy than that found using larger targets. Nevertheless, for some fairly large proteins having more than 200 residues, the low-scoring conformations sampled by FUSION reached close to the 6 Å mark. For instance, the best of five low-scoring decoys for target T0760-D1, a 210-residues β protein domain, achieved an accuracy of 6.66 Å. However, for target T0849-D1, a 236-residue mostly helical protein domain, the best of five low-scoring decoys achieved an accuracy of 8.71 Å.

**Figure 4:**
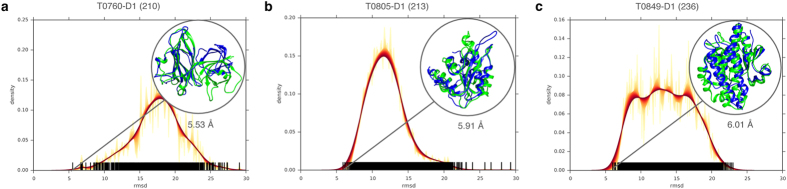
Accuracy spectrums of FUSION decoys for large proteins. (**a**–**c**) Gaussian kernel density estimation for the accuracy (Cα-rmsd) of decoys generated by FUSION for protein targets T0760-D1 (**a**), T0805-D1 (**b**), T0849-D1 (**c**), with protein size indicated within parentheses next to the target name. For each target, accuracies (C_α_-rmsd) of the whole decoy population are represented as vertical spikes along the horizontal axis, and each spike represents a decoy, along with a family of curves with varying bandwidth from 0.01 Å to 1.0 Å with a step of 0.01 Å, which corresponds to the color ramp from yellow through red. The most accurate decoys (in blue) overlaid on the experimental structures (in green) are highlighted in the insets, with their accuracies (C_α_-rmsd) specified under the insets.

To gain additional insights into the nature of the decoy population, especially for larger proteins, we examined the Gaussian kernel density estimation for the accuracy of decoys generated by FUSION. In Fig. 4, we show the distribution and degree of sampling for three targets with a sequence length of more than 200 residues. For T0760-D1 (Fig. 4a), the range of sampled conformational space is diverse with a high density of decoy population between 15  Å and 20 Å and reaching an accuracy of 5.53 Å. For T0805-D1 (Fig. 4b), the conformation space is less diverse with a definite peak near 10 Å. The best decoy attained 5.91 Å C_α_-rmsd. For T0849-D1 (Fig. 4c), the distribution is multimodal with many peaks between 6 Å and 20 Å with the best decoy reaching an accuracy of ~6 Å. The degree and distribution of conformational sampling for all targets are presented in Supplementary Fig. 2.

### Comparisons with fragment-assembly

We compared FUSION with the popular fragment assembly method ROSETTA^2^, which constructs a library of fragments from PDB using sequence profile, secondary structures, and other sequence-derived features. FUSION also assembles fragments to produce the final structure. A direct comparison between FUSION and ROSETTA, therefore, is not fair because ROSETTA has a clear advantage in its use of multiple sequence information during fragment selection. Moreover, we did not exclude homologues fragments in order to realize the full potential of ROSETTA. On the other hand, FUSION does not have such advantage since the training dataset is non-homologous to the benchmark set curated well before CASP11, and it is a model-based sampling approach rather than a fragment assembly method based on a fragment library. However, FUSION simulations used ambiguous distance restraints derived from sequence-based predicted residue-residue contacts as an additional pseudo energy term, which were not used in ROSETTA. We, therefore, decided to compare the accuracy of the best decoys generated by ROSETTA and FUSION. The comparison offers some interesting insights.

**Figure 5:**
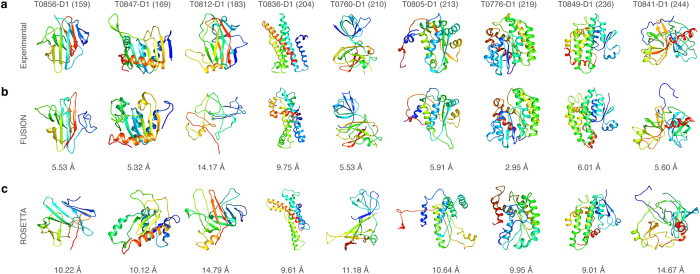
Accuracy of FUSION and ROSETTA on large targets. Ribbon diagrams of PDB files are shown for (**a**) experimental structures, (**b**) the most accurate (lowest C_α_-rmsd) decoys generated by FUSION, and **(c)** the most accurate (lowest C_α_-rmsd) decoys generated by ROSETTA for protein targets with a sequence length of more than 150 residues. All molecules are rainbow colored blue to red from the N- to C-termini. Models were optimally superimposed to the target, and then separated by translations along the vertical direction with numbers below them quantifying their accuracy (C_α_-rmsd).

Out of 16 smaller proteins with a length of less than 100 residues, ROSETTA outperformed FUSION in 14 cases in terms of the accuracy on reporting the best decoy as shown in Table 3. For instance, for target T0759-D1, a 34 residues small protein domain, the best decoy produced by ROSETTA had a C_α_-rmsd of 0.67 Å compared to the native protein, outperforming the best decoy generated by FUSION with a C_α_-rmsd of 2.38 Å by a large margin. However, for larger proteins with more than 150 residues, in terms of accuracy of the best decoy, FUSION performed better than ROSETTA. For eight out of nine targets with more than 150 residues, the best models generated by FUSION were consistently more accurate than ROSETTA. As shown in Fig. 5, in six out of nine cases, the best models generated by FUSION were reasonably accurate with a C_α_-rmsd less than 6 Å, while ROSETTA failed to reach the accuracy of 6 Å in any of the cases. Moreover, for target T0849-D1 with a sequence length of 236 residues, the best decoy generated by FUSION attained 6.01 Å C_α_-rmsd, while best decoy generated by ROSETTA had an accuracy of 9.01 Å.

## Discussion

This study introduces a probabilistic approach for sampling of a protein backbone in full atomic detail in continuous space, free from a fragment library. The sampled conformation has a reasonable stereochemistry, which is reflected by its realistic angles and secondary structure. Its ability to incorporate noisy predicted information during simulation and complete coverage of the conformational space accessible to proteins makes it fundamentally different from prior fragment assembly approaches.

An analysis of the performance of the proposed method, FUSION, in a blind assessment revealed its capability to perform convergent sampling, covering a large spectrum of conformational space accessible to a protein sequence. It performs favorably especially for larger proteins producing more accurate decoys compared to fragment assembly techniques, opening the possibility to predict near-native structural models even for large proteins in a *de novo* manner.

An obvious next step in the future is to extend the model to capture both backbone and side chain conformational bias. Given the large degrees of freedoms in the side chain of a protein molecule, this will pose a formidable computational challenge. Integrating multiple sequence alignment information into the model could be another possible direction to be investigated in the future.

To facilitate usage of the FUSION method by life scientists around the world, a public web server has been made freely available at http://protein.rnet.missouri.edu/FUSION/, where users can access and submit FUSION modeling jobs. Instructions on submitting and retrieving modeling jobs are also provided at the website. Due to limited computational resources, and to ensure a reasonable turn-around time, the maximum number of decoys per job submission is limited to 10,000.

## Methods

### Parameterization of protein conformational space

Before formulating a probabilistic model capturing detailed sequence to structure relationships, mathematical parameterization of protein conformational space is essential. Twenty naturally occurring amino acid residues usually specify protein sequence space. Due to their intrinsic stereochemistry, these residues give rise to distinct population distributions in Ramachandran space^20^. Analysis of high-resolution experimental structures^21^,^22^,^23^ has shown that it is convenient to consider these distributions in eight classes: (1) glycines not preceding prolines, (2) prolines not preceding prolines, (3) β-branched amino acid residues, isoleucines and valines, not preceding prolines, (4) all amino acids except glycines, prolines, isoleucines, and valines not preceding prolines, (5) glycines preceding prolines, (6) prolines preceding prolines, (7) β-branched residues isoleucines and valines preceding prolines, and (8) all amino acids except glycine, proline, isoleucine, and valine preceding prolines. We use these eight classes of amino acids residues to represent protein sequence space.

On the structural side, we adopt a backbone-only representation of proteins, where, each amino acid residues in a protein chain can be characterized using three angular degrees of freedom, the ϕ, ψ and ω dihedral angles, assuming ideal bond lengths and bond angles^24^. Due to the presence of steric hindrance and electrostatic interactions, backbone dihedral angle pairs (ϕ, ψ) cluster together in distinct regions of the Ramachandran plot in naturally-occurring protein structures. Densely populated regions correspond to low energy conformations found in common elements of secondary structures, most significantly, right-handed α-helices, left-handed α_L_-turns and extended β-strands. We, therefore, considered three-state secondary structure types (helix, strand and coil) to capture this preference. Furthermore, we included the peptide ω angles, which have been found to exhibit systematic variations in (ϕ, ψ) space in proteins^11^,^25^. This parameterization is simple, yet adequate to describe protein backbone conformation in atomic detail.

### Formulating the probabilistic graphical model

We briefly describe the most important aspects of the proposed model in Fig. 1. For each slice, *i*, a residue type identifier, A_*i*_ specifies which of the eight classes of residue types serves as input in a given slice, and a hidden variable, **H**, that can adopt 30 different discrete states (see below). Each of these states (H_*i*_) corresponds to a specific emission distribution over secondary structures (S_*i*_: helix, strand, and coil), dihedral angle pairs (D_*i*_: ϕ, ψ), and peptide bond conformations (P_*i*_: ω). Conformational space of a protein with *n* residues is specified by the following probability distribution:


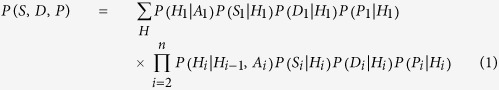


where, the sum runs over all possible hidden node sequences **H** = (H_*i*_, …, H_*n*_).

We model the discrete nodes **A** and **S** using conditional probability tables. In order to capture the angular preferences of the backbone dihedral angle pair node (**D**), we use a mixture of bivariate von Mises distributions (the cosine variant), which is most suited for this purpose^26^. Bivariate von Mises distribution specifies dihedral angle pairs (ϕ, ψ), both ranging from –π to π as points on torus. The probability density function is given by:


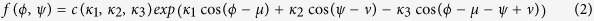


where, *μ* and *ν* are means for ϕ and ψ, respectively, *κ*_*1*_ and *κ*_*2*_ are their concentration, while *κ*_*3*_ is related to their correlation.

Angular preference of the ω dihedral angle node of a peptide bond (**P**) is modeled using a mixture of von Mises distribution^27^, which can be considered the circular equivalent of Gaussian distribution. The von Mises distribution takes the circular nature of angular data into account, but it also represents dihedral angles ranging from –π to π as points on circle. The probability density function has the following form:


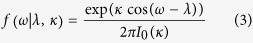


where, *λ* is the mean angle, *κ* > 0 is a concentration parameter, and *I*_*0*_ is the modified Bessel function of the first kind and of the order zero.

### Training data, parameter estimation, and model selection

As training data, we collected 1,740 non-redundant protein domains, covering different SCOP folds, from the SABmark dataset, version 1.65^28^. Residue class and angle information was extracted directly from the training data, whereas three-state secondary structures (helix, strand, and coil) were assigned using DSSP^29^. The training dataset contains 270,350 observations.

Parameter estimation for FUSION was done using Stochastic Expectation-Maximization (S-EM)^30^, as implemented in Mocapy^++^ software package^31^. In each iteration, the S-EM algorithm consisted of two steps: (1) for each observation in the training set, plausible hidden nodes were resampled using the forward-backtrack algorithm^13^, which allocated each observation in the training set to a specific hidden state (E-step); (2) the parameters were updated using maximum likelihood, assuming the model was fully observed (M-step). S-EM algorithm is known to be a better choice than classic EM algorithm on large datasets due to its computational efficiency and its ability to avoid convergence to local optima^30^.

The optimal size of the hidden node is a hyperparameter that has to be determined separately, and choosing the optimal hidden node size is crucial for the model to succeed. For low size, the model will be too coarse; however, if the size is too high, it will lead to overfitting. We estimated the optimal hidden node size using the Akaike Information Criterion (AIC)^32^, a well-established model selection criterion:


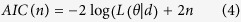


where, *L*(*θ|d*) is the likelihood of the model given the data *d*, and *n* is the number of parameters. The AIC value reaches a minimal value for the optimal model. The AIC was calculated for hidden node sizes of 10 to 100 (with a step size of 5), using a likelihood obtained after convergence of the S-EM algorithm. Since the nature of the training process is stochastic, parameter estimation for each hidden node size was repeated four times with different starting conditions. For a model with a hidden node size of 30, the AIC value reached its minimum value, resulting in 7,812 parameters (Supplementary Fig. 3). We chose this model as the optimum one.

### Conformational sampling

For a given stretch of *n* residue protein sequence, the amino acid residues can be readily mapped to the residue classes (A_*1*_, …, A_*n*_). The plausible values of the hidden nodes, H_*i*_, are then sampled from one end to the other, from the distribution *P*(*H*_*i*_|*A*_*i*_ = *a*_*i*_, *H*_*i*−1_ = *h*_*i*−1_). Based on the sequence of hidden node values, samples for corresponding emission nodes are drawn from the corresponding conditional probability distribution 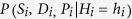.

Once we have sampled a sequence of hidden values, (H_*1*_, …, H_*n*_), a sequence of secondary structure types (S_*1*_, …, S_*n*_), a sequence of (ϕ, ψ) angle pairs (D_*1*_, …, D_*n*_), and a sequence of ω dihedral angles of the peptide bonds (P_*1*_, …, P_*n*_), given an appropriate sequence of residue classes (A_*1*_, …, A_*n*_), resampling a sub-sequence, from position *l* to *m* can then be done using the forward-backtrack algorithm^13^. The algorithm involves two steps. In the first step, the forward variables 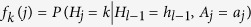 are calculated for each possible hidden node value *k* in each slice *j* ∈ (*l*, …, *m*), using the forward algorithm^33^. Subsequently, the hidden nodes values, *h*_*j*_, are sampled from position *l* to position *m* proportional to 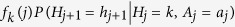. In the second step, emission nodes at each position j ∈ (*l*, …, *m*) are sampled from the conditional probability distribution 
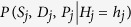
. In case the secondary structure information is available, or, predicted from the protein sequence; hence, the same sampling and resampling strategies can be applied simply by treating secondary structure types (S_*i*_) of the corresponding sequence position *i*, as observed. This unique *conditional* sampling approach makes it possible to incorporate observed structural features to guide the sampling of dihedral angles.

### Simulation protocol

For each protein in the benchmark set, we predicted a three-state secondary structure (helix, strand, and coil) from the amino acid sequence using machine-learning based secondary structure predictors PSIPRED^34^ and Raptor-X^35^. To reduce the effect of noisy prediction on the modeling performance, we flagged the secondary structure as observed only when the consensus confidence (confidence of secondary structure ∈ [0, 1]) for a residue was above 0.5. For the rest of the residues, secondary structures were left hidden, allowing flexibility during the simulations.

We used ROSETTA’s low-resolution scoring function, *E*_*rosetta*_, as one part of the FUSION’s energy functions to guide the simulations, accessed through its Python-based interface, PyRosetta^36^. Briefly, it includes terms for van der Waals hard sphere repulsion (vdw), residue environment (env), residue pair (pair), C_β_ packing density (cb), secondary structure packing [helix–helix pairing (hh), helix-strand pairing (hs), strand-strand pairing (ss), strand pair distance (rsigma) and sheet formation from strands (sheet)], plus radius of gyration (rg). The details for each of these terms have been described elsewhere^15^. In general, the ROSETTA low-resolution scoring function favors compact structures with buried hydrophobic residues, and paired β strands. To further guide the sampling, we added ambiguous distance restraints as an additional pseudo energy term using sequence-based predicted residue to residue contacts [two amino acid residues are considered to be in contact if the distance between their C_β_ atoms (C_α_ for glycine) in the experimental structure is less than 8Å] using NNcon^37^ and PhyCMAP^38^. The contact energy was defined as a function of atom pair distance restraint^39^ between C_β_ atoms (C_α_ for glycine):


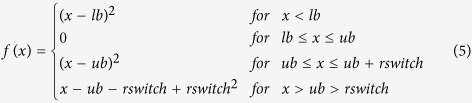


where, *x* is the distance between the corresponding atoms for a contact pair, *lb* is the lower bound (1.5 Å), *ub* is the upper bound (8 Å), and *rswitch* is a constant of 0.5. We filtered all contacts except the top *L/5* (sorted by confidence of prediction ∈ [0, 1]), and we predicted contacts from each predictor, where *L* is the sequence length of the protein. In order to further account for low accuracy in sequence-based predicted contacts, contact energy was evaluated within ± δ neighboring residues of a predicted contact pair [i, j], for small values of δ, and the minimum energy value was considered as ambiguous contact energy *E*_*ij*_ (e.g., for δ = 1, ambiguous contact energy, *E*_*ij*_, of a predicted contact pair [i, j] would be the minimum of contact energy evaluated at [i, j], [i ± 1, j], [i, j ± 1], and [i ± 1, j ± 1]). Summing up *E*_*ij*_ values over the top L/5 predicted contact pairs from each contact predictor resulted in the contact-derived restraint energy, *E*_*contact*_. The value of δ was set as a logarithmic function of the sequence separation between the residues under consideration: 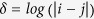. Based on our preliminary simulation runs, such an ambiguous (less than residue-level precision) definition of contact not only compensates for the noise in contact prediction, but it also facilitates achieving an optimal balance between contact-derived restraints energy and general physical chemistry, which is implicit in the ROSETTA scoring function. The total energy, *E*_*total*_ (**x**), of a conformation **x** with dihedral angle sequence **d**, is a linear combination of ROSETTA low-resolution scoring function, and contact-derived restraint energy function:


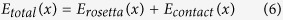


Subsequently, Boltzmann’s law was used to convert the energies into probabilities:


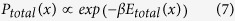


where, the inverse temperature, *β*, was set to 2.0 kT.

For a transition from a dihedral angle sequence from **d** to **d**′ in the FUSION model, Metropolis-Hastings acceptance criterion can be expressed as:


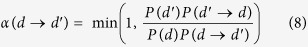


where, 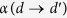 is the acceptance probability corresponding to the transition from state **d** to **d**′; moreover, *P*(**d**′) and *P*(**d**) are the probabilities of **d** and **d**′ according to the target distribution, while 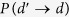 and 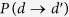 are the probabilities of a move from state **d**′ to state **d**, and vice versa, according to the proposal distribution.

Since we used FUSION as a solely proposed distribution, and the transition in dihedral sequence from state **d** to **d**′ results in a transition of conformation from **x** to **x**′, the Metropolis-Hasting expression reduces to:


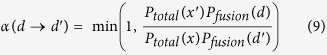


where, *P*_*total*_ (**x**) is the scoring function derived probability described above, and *P*_*fusion*_ (**d**) is the product of the probabilities of dihedral angles in **d** according to FUSION, conditioned on the residue classes and optionally secondary structure types.

We performed 20,000 MCMC iterations to generate each low-resolution model by resampling random stretches of 3 to 15 residue segment, and selected the structure with the highest probability (i.e., lowest energy). The lowest energy structure was further relaxed using a smooth reparameterized version of ROSETTA’s low-resolution scoring function^2^,^15^.

### Sources of experimental PDB structures in the benchmark set

The experimental PDB structures used in the 42-protein benchmark set were downloaded from the CASP11 website at http://predictioncenter.org/download_area/CASP11/targets/. The domain definitions and the PDB accession codes were provide by CASP assessors at http://predictioncenter.org/casp11/domains_summary.cgi.

## Additional Information

**How to cite this article**: Bhattacharya, D. and Cheng, J. *De novo* protein conformational sampling using a probabilistic graphical model. *Sci. Rep.* **5**, 16332; doi: 10.1038/srep16332 (2015).

## Supplementary Material

Supplementary Information

## Figures and Tables

**Table 1:** Secondary structure contents in the experimental structures and FUSION decoys.

Experimental structure				Lowest scoring FUSION decoy		Best of five lowest scoring FUSION decoys	
Target id^a^	Length	%α^b^	%β^c^	%α	%β	%α	%β
T0759-D1	34	47.06	11.76	50.00	0.00	55.88	0.00
T0759-D2	62	35.48	29.03	38.71	14.52	40.32	16.13
T0760-D1	210	7.46	47.76	3.98	50.25	0.00	50.25
T0763-D1	130	20.77	41.54	7.69	25.38	7.69	25.38
T0765-D1	76	31.58	31.58	31.58	23.68	31.58	10.53
T0766-D1	108	26.85	54.63	28.70	48.15	25.93	50.93
T0768-D1	143	42.27	36.08	7.69	18.18	7.69	18.18
T0769-D1	97	29.14	30.46	37.11	35.05	40.21	28.87
T0771-D1	150	40.30	31.34	15.23	31.13	19.21	10.60
T0773-D1	67	50.23	10.50	40.30	35.82	40.30	35.82
T0776-D1	219	10.00	60.91	48.86	13.24	47.95	12.33
T0782-D1	110	2.40	60.80	0.00	50.91	2.73	54.55
T0784-D1	125	8.04	59.82	12.00	47.20	2.40	54.40
T0785-D1	112	48.72	14.10	19.64	25.89	20.54	23.21
T0792-D1	78	32.84	26.12	41.03	10.26	47.44	10.26
T0803-D1	134	46.70	15.23	23.88	14.93	23.13	17.16
T0805-D1	213	14.29	41.21	38.58	13.71	38.07	14.21
T0812-D1	183	53.97	12.91	9.89	15.93	4.95	25.82
T0815-D1	106	31.13	46.23	33.02	46.23	33.02	49.06
T0816-D1	68	73.53	0.00	80.88	0.00	73.53	0.00
T0818-D1	134	32.84	41.04	28.36	16.42	32.09	15.67
T0820-D1	90	73.33	0.00	73.33	4.44	71.11	0.00
T0820-D2	36	19.44	27.78	38.89	27.78	27.78	22.22
T0822-D1	114	3.51	53.51	3.51	37.72	3.51	42.11
T0824-D1	108	37.96	25.00	29.63	14.81	32.41	14.81
T0829-D1	67	25.37	32.84	26.87	31.34	26.87	31.34
T0833-D1	108	7.41	70.37	0.00	50.93	0.00	54.63
T0834-D1	99	49.49	20.20	50.51	0.00	49.49	0.00
T0834-D2	92	60.47	2.33	36.05	0.00	50.00	0.00
T0836-D1	204	82.35	0.00	77.45	0.98	72.55	0.00
T0837-D1	121	75.21	0.00	76.03	0.00	71.90	0.00
T0838-D1	126	23.02	40.48	26.98	0.00	27.78	3.17
T0841-D1	244	9.57	25.22	11.26	35.06	11.26	35.06
T0847-D1	169	33.14	30.77	31.95	19.53	33.73	21.30
T0849-D1	236	60.17	8.05	58.05	7.63	56.36	7.63
T0853-D1	76	15.79	31.58	17.11	19.74	22.37	10.53
T0853-D2	72	25.00	37.50	26.39	20.83	26.39	20.83
T0854-D1	132	36.36	21.21	41.67	19.70	43.18	18.94
T0854-D2	70	68.57	0.00	67.14	5.71	67.14	5.71
T0855-D1	115	33.91	24.35	34.78	28.70	34.78	28.70
T0856-D1	159	0.00	50.31	1.89	37.11	1.89	45.28
T0857-D1	96	0.00	52.08	0.00	40.63	5.21	41.67
Average	121^d^	33.94	29.92	31.59	22.37	31.68	21.60

^a^Identification numbers of the targets assigned by CASP11 assessors.

^b^Percentage of residues in alpha helix.

^c^Percentage of residues in beta strand.

^d^Rounded to nearest integer.

**Table 2:** Accuracy of FUSION decoys.

Target id^a^	Length	C_α_-rmsd (Å)		
		<6 Å (Percent)	Lowest Scoring	Best of five lowest scoring
**T0759-D1**	**34**	**16.67**	**6.52**	**5.92**
**T0759-D2**	**62**	**0.98**	**11.39**	**4.62**
**T0760-D1**	**210**	**0.11**	**6.86**	**6.66**
T0763-D1	130	0.00	15.86	15.86
**T0765-D1**	**76**	**20.85**	**4.32**	**4.23**
**T0766-D1**	**108**	**2.85**	**2.95**	**2.64**
T0768-D1	143	0.00	9.41	9.41
**T0769-D1**	**97**	**55.73**	**3.99**	**3.17**
T0771-D1	150	0.00	17.45	17.22
**T0773-D1**	**67**	**62.76**	**2.19**	**2.19**
**T0776-D1**	**219**	**52.60**	**3.56**	**3.42**
T0782-D1	110	0.00	8.86	8.35
**T0784-D1**	**125**	**0.05**	**13.73**	**5.32**
T0785-D1	112	0.00	14.46	10.48
**T0792-D1**	**78**	**0.51**	**10.44**	**8.47**
T0803-D1	134	0.00	16.37	12.18
**T0805-D1**	**213**	**0.03**	**11.04**	**8.46**
T0812-D1	183	0.00	19.30	17.37
**T0815-D1**	**106**	**72.26**	**3.36**	**2.44**
**T0816-D1**	**68**	**38.32**	**7.78**	**3.63**
T0818-D1	134	0.00	12.27	8.70
**T0820-D1**	**90**	**0.01**	**12.86**	**12.07**
**T0820-D2**	**36**	**51.36**	**6.04**	**4.23**
T0822-D1	114	0.00	13.89	11.78
T0824-D1	108	0.00	14.19	12.25
**T0829-D1**	**67**	**9.07**	**3.85**	**3.85**
**T0833-D1**	**108**	**0.79**	**7.12**	**6.87**
T0834-D1	99	0.00	16.18	11.16
T0834-D2	92	0.00	13.53	11.82
T0836-D1	204	0.00	15.22	14.65
**T0837-D1**	**121**	**0.02**	**15.42**	**10.11**
T0838-D1	126	0.00	17.49	12.50
**T0841-D1**	**244**	**0.05**	**6.28**	**6.28**
**T0847-D1**	**169**	**0.32**	**6.48**	**5.55**
T0849-D1	236	0.00	12.16	8.71
T0853-D1	76	0.00	14.19	10.30
T0853-D2	72	0.00	9.39	9.39
**T0854-D1**	**132**	**60.62**	**2.04**	**1.80**
**T0854-D2**	**70**	**59.19**	**2.62**	**2.62**
**T0855-D1**	**115**	**0.05**	**8.99**	**8.99**
**T0856-D1**	**159**	**0.04**	**9.95**	**7.37**
T0857-D1	96	0.00	12.58	10.93

^a^Targets highlighted in bold indicates that FUSION generated some decoys with Cα-rmsd (Å) less than <6 Å.

**Table 3:** Accuracy of best decoys generated by FUSION and ROSETTA.

Target id^a^	Length	C_α_-rmsd (Å)	
		FUSION	ROSETTA
T0759-D1	34	2.38	0.67
T0759-D2	62	3.69	2.79
**T0760-D1**	**210**	**5.53**	**11.18**
T0763-D1	130	11.32	10.52
T0765-D1	76	3.42	1.96
T0766-D1	108	2.64	4.62
T0768-D1	143	7.68	5.78
T0769-D1	97	3.02	2.34
T0771-D1	150	11.75	9.87
T0773-D1	67	1.97	0.97
**T0776-D1**	**219**	**2.95**	**9.95**
T0782-D1	110	7.29	4.21
T0784-D1	125	3.49	12.43
T0785-D1	112	10.00	9.33
T0792-D1	78	4.07	3.50
T0803-D1	134	9.33	7.72
**T0805-D1**	**213**	**5.91**	**10.64**
**T0812-D1**	**183**	**14.17**	**14.79**
T0815-D1	106	1.94	2.63
T0816-D1	68	2.10	1.76
T0818-D1	134	7.84	9.09
T0820-D1	90	5.98	5.70
T0820-D2	36	2.56	2.02
T0822-D1	114	9.40	9.68
T0824-D1	108	7.90	7.86
T0829-D1	67	3.15	1.87
T0833-D1	108	3.62	3.06
T0834-D1	99	11.05	9.82
T0834-D2	92	7.70	7.06
**T0836-D1**	**204**	**9.75**	**9.61**
T0837-D1	121	5.85	3.04
T0838-D1	126	8.80	8.74
**T0841-D1**	**244**	**5.60**	**14.67**
**T0847-D1**	**169**	**5.32**	**10.12**
**T0849-D1**	**236**	**6.01**	**9.01**
T0853-D1	76	7.57	5.21
T0853-D2	72	7.18	4.81
T0854-D1	132	1.76	4.57
T0854-D2	70	2.31	3.52
T0855-D1	115	5.52	4.18
**T0856-D1**	**159**	**5.53**	**10.22**
T0857-D1	96	8.76	8.77

^a^Targets highlighted in bold have sequence length of more than 150 residues.

## References

[b1] Levinthal C. Are there pathways for protein folding. J. Chim. phys 65, 44–45 (1968).

[b2] Simons K. T., Kooperberg C., Huang E. & Baker D. Assembly of protein tertiary structures from fragments with similar local sequences using simulated annealing and Bayesian scoring functions. J. Mol. Biol. 268, 209–225 (1997). 9149153 10.1006/jmbi.1997.0959

[b3] Chikenji G., Fujitsuka Y. & Takada S. A reversible fragment assembly method for *de novo* protein structure prediction. The Journal of Chemical Physics 119, 6895–6903 (2003).

[b4] Chikenji G., Fujitsuka Y. & Takada S. Shaping up the protein folding funnel by local interaction: lesson from a structure prediction study. Proc. Natl. Acad. Sci. USA 103, 3141–3146 (2006). 16488978 10.1073/pnas.0508195103 PMC1413881

[b5] Bradley P., Misura K. M. & Baker D. Toward high-resolution *de novo* structure prediction for small proteins. Science 309, 1868–1871 (2005). 16166519 10.1126/science.1113801

[b6] Hegler J. A., Lätzer J., Shehu A., Clementi C. & Wolynes P. G. Restriction versus guidance in protein structure prediction. Proc. Natl. Acad. Sci. 106, 15302–15307 (2009). 19706384 10.1073/pnas.0907002106 PMC2741246

[b7] Kim D. E., Blum B., Bradley P. & Baker D. Sampling bottlenecks in *de novo* protein structure prediction. J. Mol. Biol. 393, 249–260 (2009). 19646450 10.1016/j.jmb.2009.07.063 PMC2760740

[b8] Hamelryck T., Kent J. T. & Krogh A. Sampling realistic protein conformations using local structural bias. PLoS Comput. Biol. 2, e131 (2006). 17002495 10.1371/journal.pcbi.0020131 PMC1570370

[b9] Zhao F., Li S., Sterner B. W. & Xu J. Discriminative learning for protein conformation sampling. Proteins: Structure, Function, and Bioinformatics 73, 228–240 (2008). 10.1002/prot.22057 PMC2826217 18412258

[b10] Boomsma W. *et al.* A generative, probabilistic model of local protein structure. Proc. Natl. Acad. Sci. 105, 8932–8937 (2008). 18579771 10.1073/pnas.0801715105 PMC2440424

[b11] Berkholz D. S., Driggers C. M., Shapovalov M. V., Dunbrack R. L. & Karplus P. A. Nonplanar peptide bonds in proteins are common and conserved but not biased toward active sites. Proc. Natl. Acad. Sci. 109, 449–453 (2012). 22198840 10.1073/pnas.1107115108 PMC3258596

[b12] Bengio Y. & Frasconi P. Input-output HMMs for sequence processing. Neural Networks, IEEE Transactions on 7, 1231–1249 (1996). 10.1109/72.536317 18263517

[b13] Cawley S. L. & Pachter L. HMM sampling and applications to gene finding and alternative splicing. Bioinformatics 19, ii36–ii41 (2003). 14534169 10.1093/bioinformatics/btg1057

[b14] Gilks W. R., Richardson S. & Spiegelhalter D.J. Introducing markov chain monte carlo. Markov chain Monte Carlo in practice 1, 19 (1996).

[b15] Rohl C. A., Strauss C. E., Misura K. M. & Baker D. Protein structure prediction using Rosetta. Methods Enzymol. 383, 66–93 (2004). 15063647 10.1016/S0076-6879(04)83004-0

[b16] Przytycka T. Significance of conformational biases in Monte Carlo simulations of protein folding: Lessons from Metropolis–Hastings approach. Proteins: Structure, Function, and Bioinformatics 57, 338–344 (2004). 10.1002/prot.20210 15340921

[b17] Shapovalov M. V. & Dunbrack R. L. A smoothed backbone-dependent rotamer library for proteins derived from adaptive kernel density estimates and regressions. Structure 19, 844–858 (2011). 21645855 10.1016/j.str.2011.03.019 PMC3118414

[b18] Kuhlman B. & Baker D. Native protein sequences are close to optimal for their structures. Proc. Natl. Acad. Sci. 97, 10383–10388 (2000). 10984534 10.1073/pnas.97.19.10383 PMC27033

[b19] Zhou H. & Zhou Y. Distance‐scaled, finite ideal‐gas reference state improves structure‐derived potentials of mean force for structure selection and stability prediction. Protein Sci. 11, 2714–2726 (2002). 12381853 10.1110/ps.0217002 PMC2373736

[b20] Ramachandran G., Ramakrishnan C. & Sasisekharan V. Stereochemistry of polypeptide chain configurations. J. Mol. Biol. 7, 95–99 (1963). 13990617 10.1016/s0022-2836(63)80023-6

[b21] Lovell S. C. *et al.* Structure validation by Cα geometry: ϕ, ψ and Cβ deviation. Proteins: Structure, Function, and Bioinformatics 50, 437–450 (2003). 10.1002/prot.10286 12557186

[b22] Ho B. K. & Brasseur R. The Ramachandran plots of glycine and pre-proline. BMC Struct. Biol. 5, 14 (2005). 16105172 10.1186/1472-6807-5-14 PMC1201153

[b23] Karplus P. A. Experimentally observed conformation-dependent geometry and hidden strain in proteins. Protein Sci. 5, 1406–1420 (1996). 8819173 10.1002/pro.5560050719 PMC2143451

[b24] Engh R. A. & Huber R. Accurate bond and angle parameters for X-ray protein structure refinement. Acta Crystallographica Section A: Foundations of Crystallography 47, 392–400 (1991).

[b25] MacArthur M. W. & Thornton J. M. Deviations from planarity of the peptide bond in peptides and proteins. J. Mol. Biol. 264, 1180–1195 (1996). 9000639 10.1006/jmbi.1996.0705

[b26] Mardia K. V., Taylor C. C. & Subramaniam G. K. Protein bioinformatics and mixtures of bivariate von Mises distributions for angular data. Biometrics 63, 505–512 (2007). 17688502 10.1111/j.1541-0420.2006.00682.x

[b27] Mardia K. V. & Jupp P. E. Directional Statistics. Vol. 494 (John Wiley & Sons, 2009).

[b28] Van Walle I., Lasters I. & Wyns L. SABmark—a benchmark for sequence alignment that covers the entire known fold space. Bioinformatics 21, 1267–1268 (2005). 15333456 10.1093/bioinformatics/bth493

[b29] Kabsch W. & Sander C. Dictionary of protein secondary structure: pattern recognition of hydrogen-bonded and geometrical features. Biopolymers 22, 2577–2637 (1983). 6667333 10.1002/bip.360221211

[b30] Nielsen S. F. The stochastic EM algorithm: estimation and asymptotic results. Bernoulli, 457–489 (2000).

[b31] Paluszewski M. & Hamelryck T. Mocapy++-A toolkit for inference and learning in dynamic Bayesian networks. BMC Bioinformatics 11, 126 (2010). 20226024 10.1186/1471-2105-11-126 PMC2848649

[b32] Burnham K. P. & Anderson D. R. Model Selection and Multimodel Inference: a Practical Information-Theoretic Approach. (Springer Science & Business Media, 2002).

[b33] Durbin R. Biological Sequence Analysis: Probabilistic Models of Proteins and Nucleic Acids. (Cambridge university press, 1998).

[b34] Jones D. T. Protein secondary structure prediction based on position-specific scoring matrices. J. Mol. Biol. 292, 195–202 (1999). 10493868 10.1006/jmbi.1999.3091

[b35] Wang Z., Zhao F., Peng J. & Xu J. Protein 8-class secondary structure prediction using conditional neural fields. Proteomics 11, 3786–3792 (2011). 21805636 10.1002/pmic.201100196 PMC3341732

[b36] Chaudhury S., Lyskov S. & Gray J. J. PyRosetta: a script-based interface for implementing molecular modeling algorithms using Rosetta. Bioinformatics 26, 689–691 (2010). 20061306 10.1093/bioinformatics/btq007 PMC2828115

[b37] Tegge A. N., Wang Z., Eickholt J. & Cheng J. NNcon: improved protein contact map prediction using 2D-recursive neural networks. Nucleic Acids Res. 37, W515–W518 (2009). 19420062 10.1093/nar/gkp305 PMC2703959

[b38] Wang Z. & Xu J. Predicting protein contact map using evolutionary and physical constraints by integer programming. Bioinformatics 29, i266–i273 (2013). 23812992 10.1093/bioinformatics/btt211 PMC3694661

[b39] Raman S. *et al.* NMR structure determination for larger proteins using backbone-only data. Science 327, 1014–1018 (2010). 20133520 10.1126/science.1183649 PMC2909653

