# Supplementary material for: De novo protein conformational sampling using a probabilistic graphical model
Source: Sci Rep. 2015 Nov 6;5:16332. doi: 10.1038/srep16332 (PMC4635387; doi:10.1038/srep16332)

**Supplementary Information** for

“De novo protein conformational sampling using a probabilistic graphical model”

Debswapna Bhattacharya^1^ and Jianlin Cheng^1, 2, 3, *^

^1^Department of Computer Science, University of Missouri, Columbia, MO 65211, USA

^2^Informatics Institute, University of Missouri, Columbia, MO 65211, USA

^3^Bond Life Science Center, University of Missouri, Columbia, MO 65211, USA

^*^To whom correspondence should be addressed. Phone: (573)-882-7306. Fax: (573)-882-8318. E-mail: chengji@missouri.edu.

| **Supplementary Item** | **Title** |
| --- | --- |
| Supplementary Figure 1 | Energy landscapes of FUSION simulations. |
| Supplementary Figure 2 | Distribution of accuracy for FUSION decoy population. |
| Supplementary Figure 3 | Training FUSION IOHMM and model selection. |

**Supplementary Figure 1. Energy landscapes of FUSION simulations**. DFIRE score verses C_α_-rmsd for decoys produced by FUSION over the whole benchmark set (target name specified above) are shown, with color code from blue to red for low to high density, a measure of underlying energy scale.


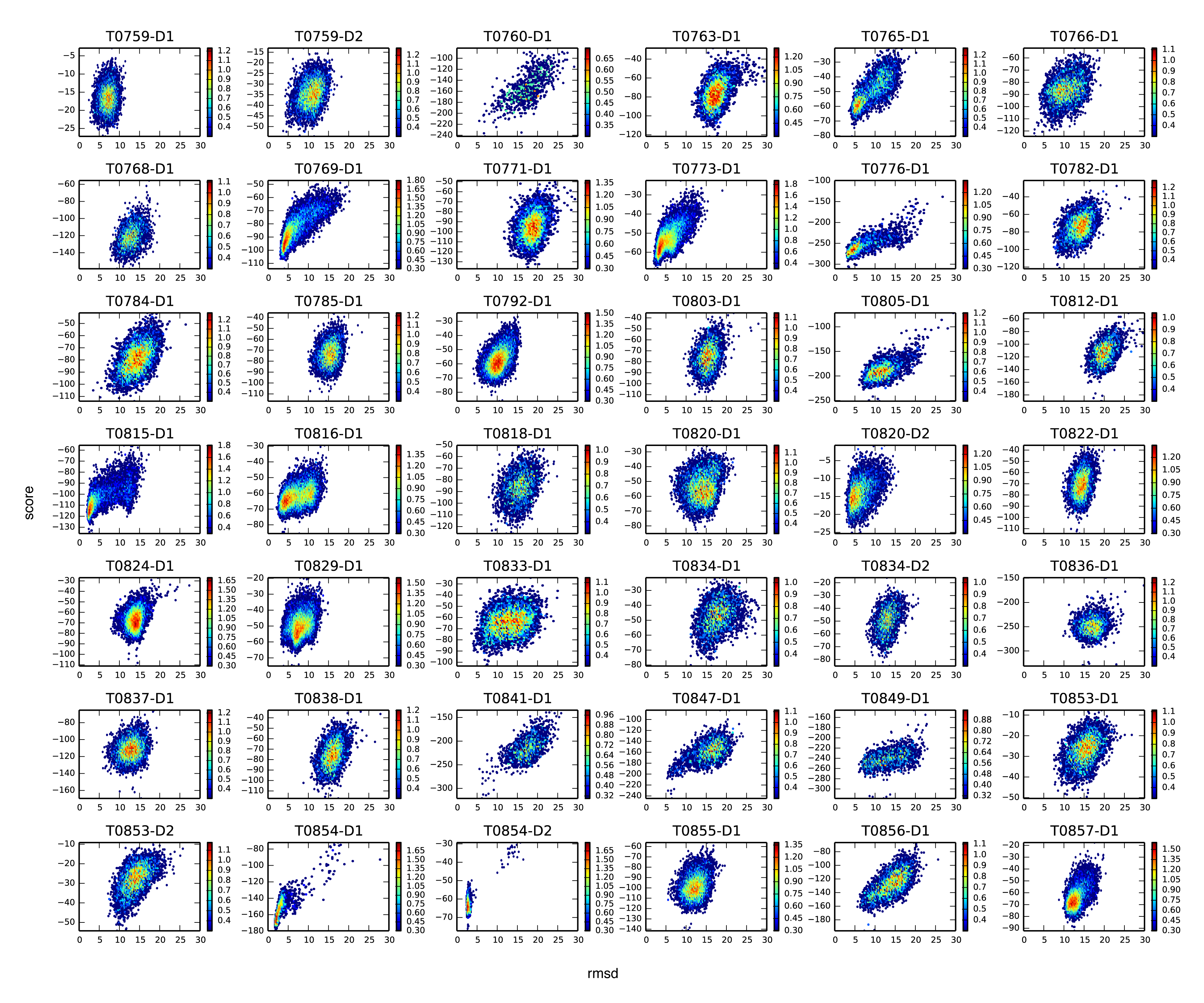


**Supplementary Figure 2. Distribution of accuracy for FUSION decoy population.** Gaussian kernel density estimation for the accuracy (C_α_-rmsd) of decoys generated by FUSION, with target name indicated above. For each target, accuracies (C_α_-rmsd) of the whole decoy population are represented as vertical spikes along the horizontal axis, each spike representing a decoy, along with the family of curves with varying bandwidth from 0.01 Å to 1.0 Å with a step of 0.01 Å, which corresponds to the color ramp from yellow through red.


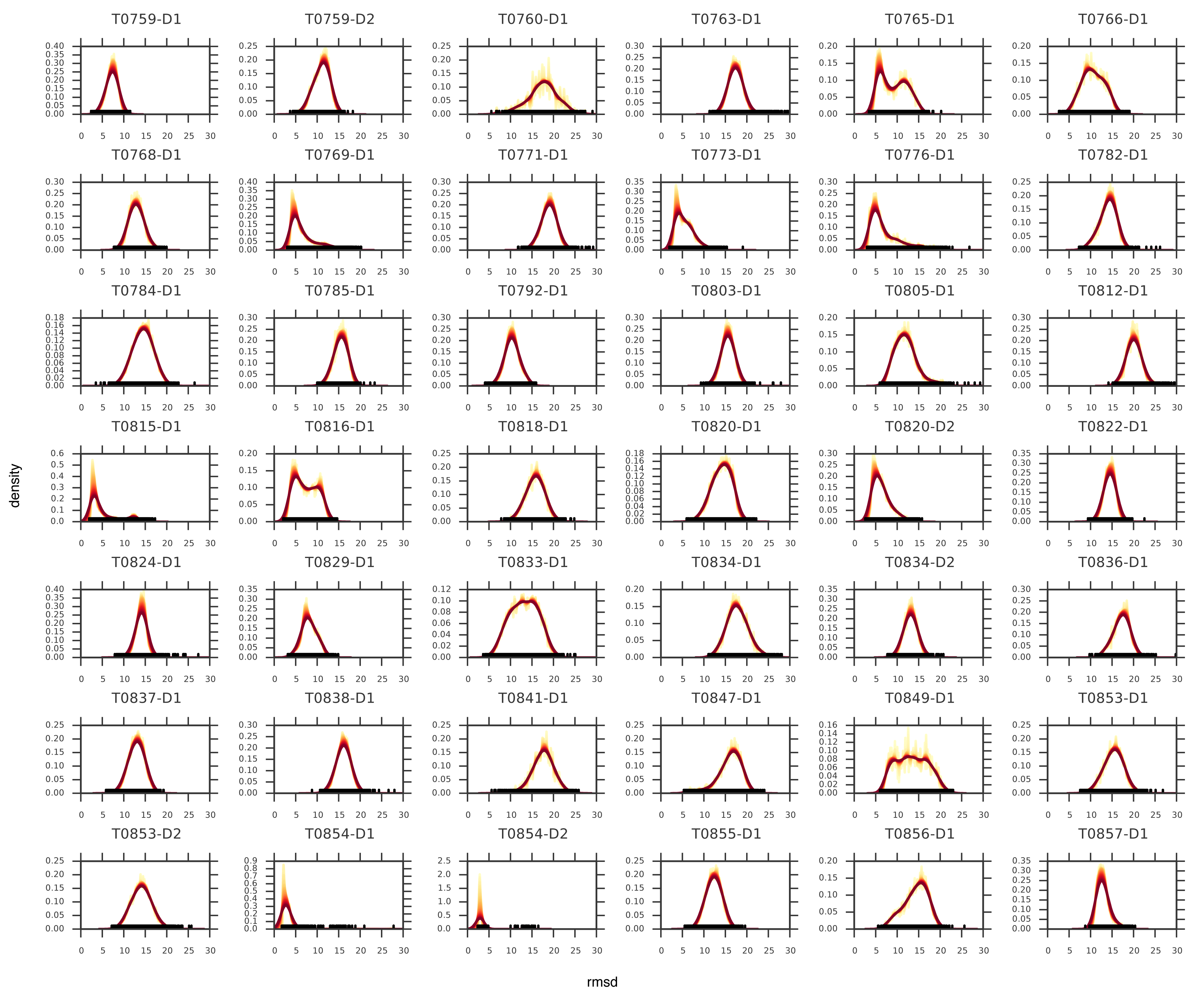


**Supplementary Figure 3. Training FUSION IOHMM and model selection.** (**a**) AIC values verses varying hidden node sizes are shown, with four models trained for each hidden node size. The curved line is tendency line constructed by fitting sixth degree polynomial to the data. The minimum AIC value corresponds to the optimal model (highlighted in red circle). (**b**) Convergence of log likelihood of the completed data during training is shown with respect to the number of S-EM iterations.


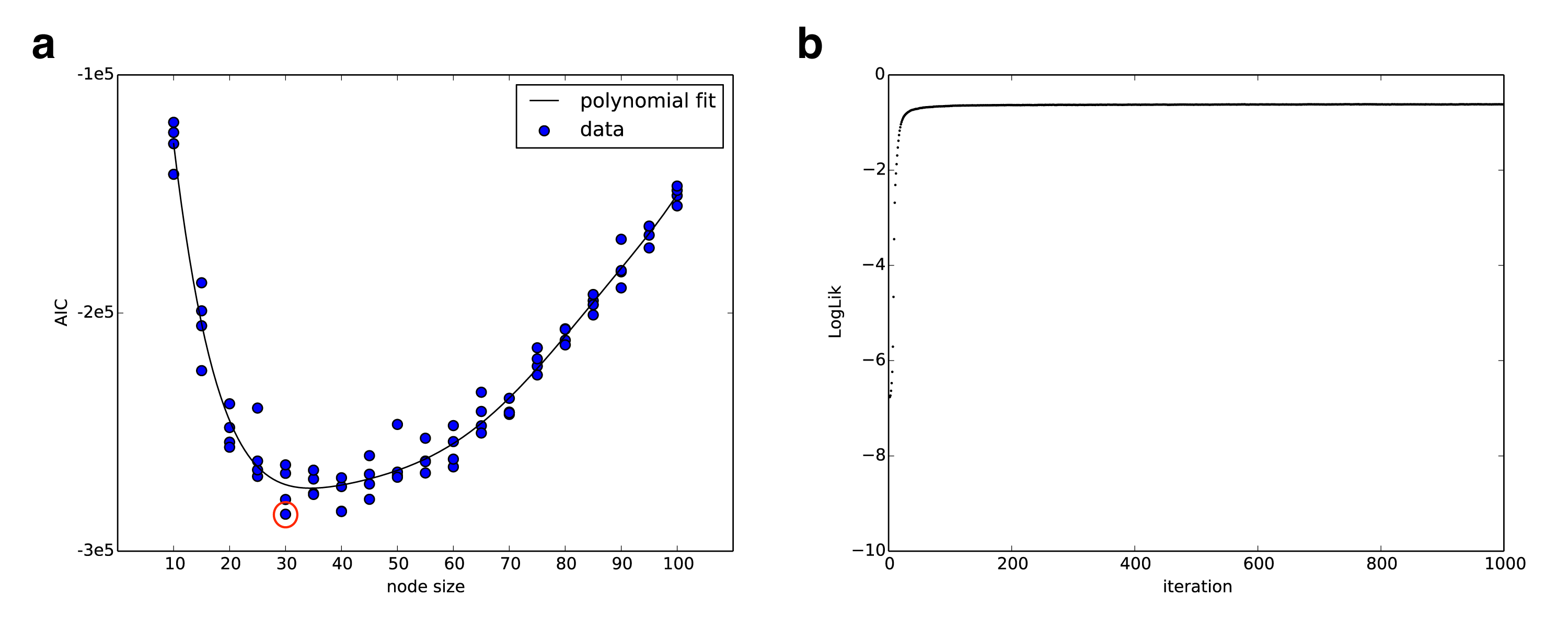

Supplement: Supplementary Information [file srep16332-s1.doc]
